# Novel insights into the immune cell landscape and gene signatures in autism spectrum disorder by bioinformatics and clinical analysis

**DOI:** 10.3389/fimmu.2022.1082950

**Published:** 2023-01-25

**Authors:** Hongwei Li, Yiran Xu, Wenhua Li, Lingling Zhang, Xiaoli Zhang, Bingbing Li, Yiwen Chen, Xiaoyang Wang, Changlian Zhu

**Affiliations:** ^1^ Henan Key Laboratory of Child Brain Injury and Henan Pediatric Clinical Research Center, Institute of Neuroscience and the Third Affiliated Hospital of Zhengzhou University, Zhengzhou, China; ^2^ National Health Council (NHC) Key Laboratory of Birth Defects Prevention, Henan Key Laboratory of Population Defects Prevention, Zhengzhou, China; ^3^ Centre of Perinatal Medicine and Health, Institute of Clinical Science, University of Gothenburg, Gothenburg, Sweden; ^4^ Center for Brain Repair and Rehabilitation, Institute of Neuroscience and Physiology, University of Gothenburg, Gothenburg, Sweden

**Keywords:** autism spectrum disorder, whole blood, immune cells landscape, predictor, bioinformatics analysis

## Abstract

The pathogenesis of autism spectrum disorder (ASD) is not well understood, especially in terms of immunity and inflammation, and there are currently no early diagnostic or treatment methods. In this study, we obtained six existing Gene Expression Omnibus transcriptome datasets from the blood of ASD patients. We performed functional enrichment analysis, PPI analysis, CIBERSORT algorithm, and Spearman correlation analysis, with a focus on expression profiling in hub genes and immune cells. We validated that monocytes and nonclassical monocytes were upregulated in the ASD group using peripheral blood (30 children with ASD and 30 age and sex-matched typically developing children) using flow cytometry. The receiver operating characteristic curves (*PSMC4* and *ALAS2*) and analysis stratified by ASD severity (*LIlRB1* and *CD69*) showed that they had predictive value using the “training” and verification groups. Three immune cell types – monocytes, M2 macrophages, and activated dendritic cells – had different degrees of correlation with 15 identified hub genes. In addition, we analyzed the miRNA-mRNA network and agents-gene interactions using miRNA databases (starBase and miRDB) and the DSigDB database. Two miRNAs (miR-342-3p and miR-1321) and 23 agents were linked with ASD. These findings suggest that dysregulation of the immune system may contribute to ASD development, especially dysregulation of monocytes and monocyte-derived cells. ASD-related hub genes may serve as potential predictors for ASD, and the potential ASD-related miRNAs and agents identified here may open up new strategies for the prevention and treatment of ASD.

## Introduction

Autism spectrum disorder (ASD) is a group of disorders characterized by impairment in social interaction and communication and the presence of restricted and repetitive behaviors and interests (1). It is often combined with intellectual disability (2), immune dysfunction (3), and inflammatory diseases of the gastrointestinal tract (4). ASD is a multifactorial process and is associated with an adverse maternal environment, children’s lifestyle, genetic factors, and immune, inflammatory, and psychosocial factors (5–8). Among these, immune and inflammatory factors have been shown to play an essential role in the development of autism (9–12). However, the pathogenesis caused by immune responses and inflammation remains unclear, and the regulatory mechanisms still require in-depth investigations.

Recent studies have shown that immune cells play a vital role in the occurrence and development of ASD. For example, abnormalities in the peripheral blood monocytes of the innate immune system are associated with behavioral changes in inflammatory subtype ASD (13), while NK cells from patients with high-functioning ASD show a high level of cell activation (14). Myeloid dendritic cells are increased in autistic children and are related to amygdala volume and repetitive behaviors (15). Microglia, as the resident macrophages in the central nervous system in persons with ASD, show elevated protein synthesis that causes autism-like synaptic and behavioral aberrations (16), and mediators from mast cells can activate microglia causing local inflammation and contributing to ASD symptoms (17). Moreover, regulatory B cells and T cells are decreased in children with ASD, and this plays a pivotal role in the evolution and severity of ASD (18). However, no study has analyzed the immune cell landscape in whole blood in persons with ASD and there is a need for a systematic approach to assessing the contribution of immune cells and key immune-related genes.

Non-coding RNAs with post-transcriptional regulatory functions for genes have been shown to be closely associated with ASD. For instance, the blood transcriptome in persons with ASD shows co-expression modules associated with ASD risk genes, including genes related to metabolism, immunity, neurodevelopment, and signal transduction (19). A recent study showed that micro-RNA (miRNA) is the key regulator of gene expression in neurodevelopmental transcriptional networks, and circulating miRNAs might therefore be potential predictors for ASD diagnosis and prognosis (20). In addition, many studies have focused on blood and saliva samples of ASD patients, and the results have shown that miRNAs might be of predictive significance in ASD because of their associations with inflammation and immunity (21–23). However, although miRNAs show great potential in treating cancer and other diseases (24), few studies on ASD have been reported. Also, the effects of known genes on ASD have not yet been fully elucidated, and more gene sets should be concentrated on, and potential interactions between genes might be further constructed and investigated such as miRNA-mRNA networks and protein-protein interaction (PPI) networks.

Bioinformatics analysis has attracted much attention with continuous breakthroughs in the discovery of novel genes and predictors. The purpose of this study was to collect and analyze the available ASD Gene Expression Omnibus (GEO) database to identify ASD-related immune cells and hub genes related to biological functions, gene networks, diagnosis, and treatment.

## Materials and methods

### Data collection and processing

**Figure 1:**
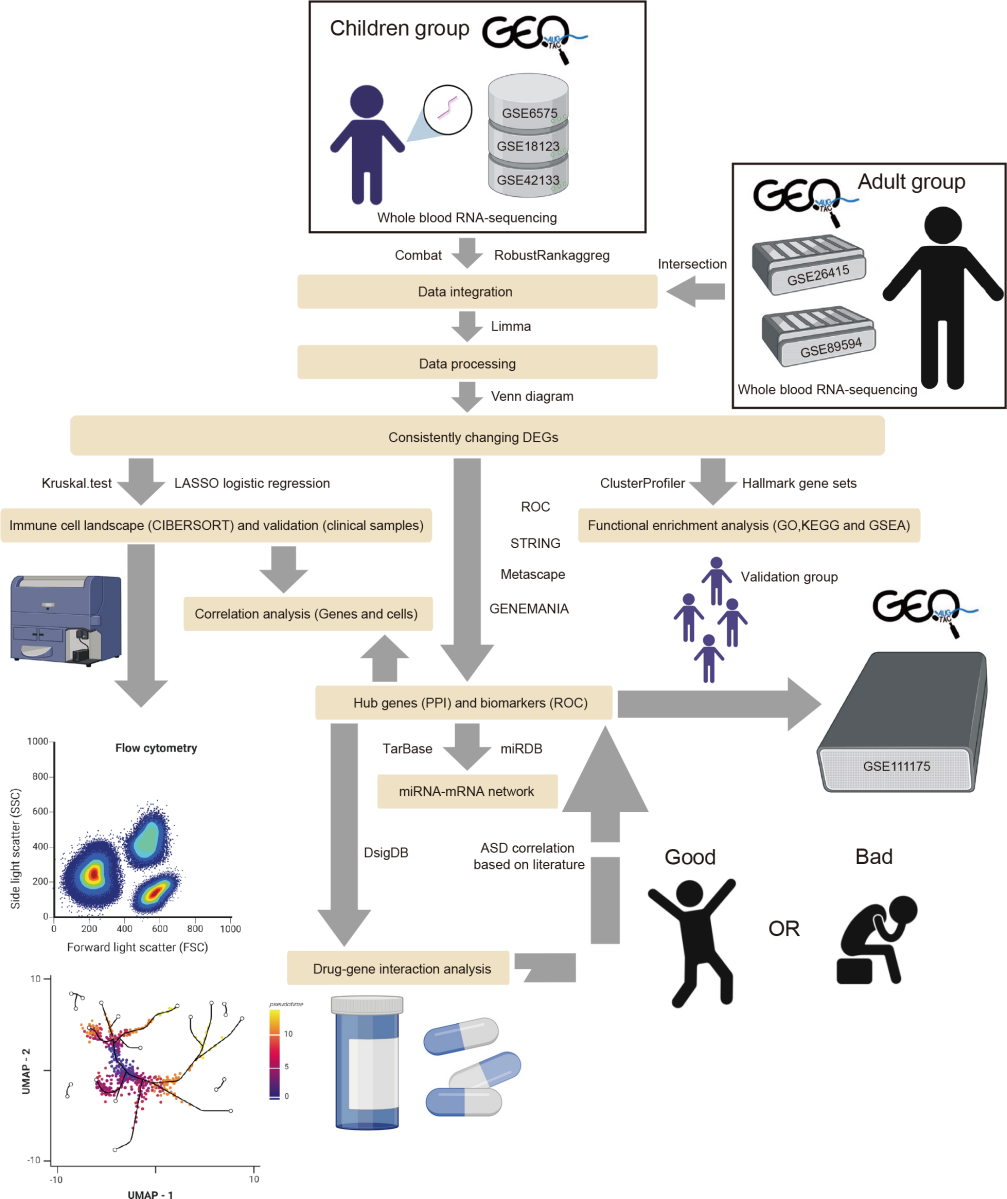
The flow diagram of the study. The adult group (GSE26415 and GSE89594) was only analyzed regarding GO, KEGG, GSEA, and immune cell infiltration.

To collect all existing datasets that used children and adults with ASD’s peripheral blood for transcriptome studies, we searched the datasets from the GEO database (https://www.ncbi.nlm.nih.gov/geo/) with the condition terms “Autism spectrum disorder,” “Autism,” “Autistic disorder,” “ASD,” the tissue term “blood,” and the organism term “Homo Sapiens.” The inclusion criteria were as follows: the datasets contained gene expression profiling by microarray or high-throughput RNA sequencing, the datasets included ASD and typically developing (TD) samples, and 20 or more samples were in the dataset. Thus, six eligible GEO Series (GSE) datasets were adopted, including GSE6575 (25), GSE18123 (26), GSE42133 (27), GSE111175 (28), GSE26415 (29), and GSE89594 (30). All transcriptomic datasets are listed in **Supplementary Table 1**. The GSE18123 dataset consisted of two RNA sequencing profiles from different GEO Platforms (GPL), so there were five datasets from children and two from adults. Among the five children’s datasets, four were used as “training” datasets, and one was used as the validation dataset. **Figure 1** illustrates the workflow of this study. The R package “limma” (v3.50.3) was used to analyze each gene expression matrix with the threshold of *p* < 0.05 (31).

### Functional and signal pathway enrichment analysis

The biological functions and corresponding pathways of the identified DEGs were determined through Gene Ontology (GO) and Kyoto Encyclopedia of Genes and Genomes (KEGG) analyses using the R package “clusterProfiler” (32) (v4.2.2) with a significance level of *p_adjusted_* < 0.05. The online databases GENEMANIA (https://genemania.org/) (33) and Metascape (http://metascape.org/gp/index.html#/main/step1) (34) were used to predict gene function. Gene Set Enrichment Analysis (GSEA) was conducted in R software using the hallmarks gene set “h.all.v7.5.1.symbols.gm” (35). The results were visualized with the R packages “enrichplot” (v1.14.2) and “ggplot2” (v3.3.6).

### Analysis of the immune cell landscape

The CIBERSORT analytical tool (https://cibersortx.stanford.edu/) was used to evaluate the abundance and differences of 22 types of immune cells between the ASD and TD group (36), including different kinds of B cells, dendritic cells, macrophages, mast cells, NK cells, T cells, eosinophils, monocytes, neutrophils, and plasma cells. The results were analyzed using the R packages “ggplot2” and “Kruskal.test”. For the training dataset, LASSO logistic regression was carried out to further select significantly different immune cells in combination with Kruskal.test. The Wilcoxon test and receiver operating characteristic (ROC) curves were calculated using R-based tools to assess the classifier performance for the LASSO regression model (v4.1.0).

### PPI analysis and hub gene screening

A PPI network was constructed among the consistently changing DEGs using the STRING database (https://string-db.org) (37). The CytoHubba module in Cytoscape (v 3.7.1) was used to score the top 10 node genes using 12 different algorithms, namely MCC (Maximum Clique Centrality), DMNC (Density of Maximum Neighborhood Component), MNC (Maximum Neighborhood Component), Degree, EPC (Edge Percolated Component), BottleNeck, EcCentricity, Closeness, Radiality, Betweenness, Stress, and Clustering Coefficient. The hub genes generated from each algorithm were shown by the R package “UpSet” and validated using GSE111175.

### Validation of significant immune cells

To validate the most significant immune cells in children, the whole blood samples from 30 children with ASD and 30 age and sex-matched TD children were collected at the 3rd Affiliated Hospital of Zhengzhou University. All participants provided informed consent to participate in the study, which was approved by the Medical Ethics Committee of the 3rd Affiliated Hospital of Zhengzhou University (Ethical number 2020–56). All blood samples were processed within 8 h for flow cytometry with a mix of antibodies according to the manufacturer’s instructions. The antibodies purchased from BD Biosciences included CD45 (HI30, Cat# 564105) CD14 (M5E2, Cat# 561712), CD16 (3G8, Cat# 563692), and HLA-DR (G46-6, Cat# 560896). The data were analyzed using FlowJo software (TreeStar) and presented using the UMAP method.

### Correlation analysis between hub genes and significantly related immune cells

The association of the hub genes with the infiltration levels of immune cells was explored and analyzed in R, and the correlation results were visualized using the “ggplot2” and “ggstatsplot” packages.

### Analysis of the predictive value of predictors

The GSE111175 dataset was used to assess the predictive effectiveness according to the validation group between the children with ASD and the children in the TD group using ROC curve analysis. The results were visualized with the “pROC”, “ggplot2”, and “ComplexHeatmap” packages.

### Construction of the mRNA-miRNA regulatory network

The starBase (predicted program ≥ 2) (38) and miRDB (39) databases were used to predict the upstream miRNAs of hub mRNAs by taking intersections. Cytoscape (v 3.7.1) was used to visualize the mRNA-miRNA network.

### Prediction of potential agents affecting ASD

The DSigDB database (http://tanlab.ucdenver.edu/DSigDB) was used to predict potential agents for hub genes related to ASD, with agents–hub genes ≥3 as the condition for the agents screen. The results were displayed using a Sankey diagram based on the “ggalluvial” (v0.12.3) package.

### Statistical analysis

All data from the bioinformatics analyses were analyzed in R, and the data from routine blood tests were analyzed using SPSS software version 23 (IBM Corp). All data are presented as the mean ± standard deviation.

## Results

### Screening of DEGs between ASD and TD in child and adult datasets

**Figure 2:**
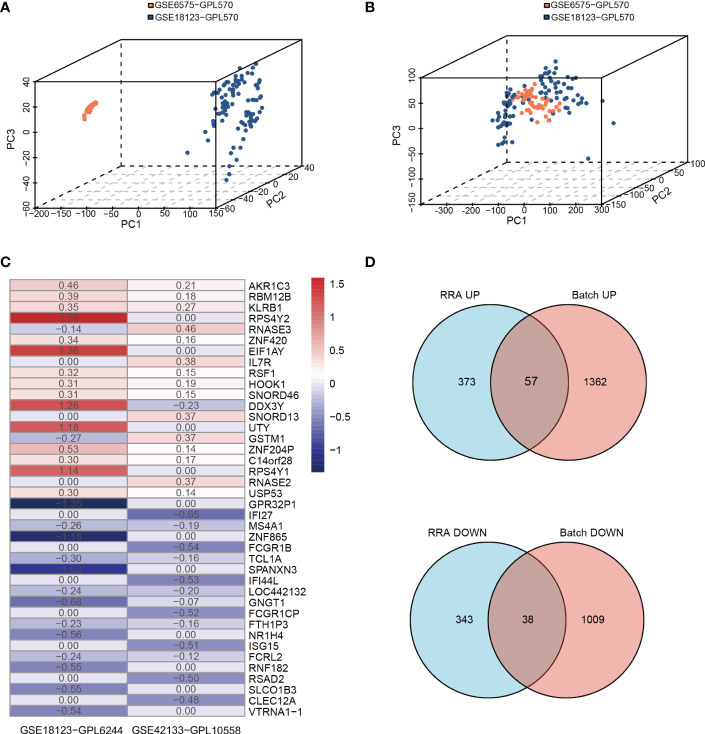
Identifying the DEGs from two methods in children’s databases. **(A, B)** Principle component analysis of the batch correction of GSE6575 and GSE18123: **(A)** before batch correction and **(B)** after batch correction. **(C)** The top 20 upregulated and 20 downregulated DEGs of the different platforms from two datasets determined by “RRA”. **(D)** Venn diagram showing the consistently changing DEGs screened by “RRA” and “Batch”.

We conducted the analysis using the four children’s datasets and two adults’ datasets. For the children’s datasets, the “RRA” and “Batch correction” methods were used to get accurate DEGs from multiple children’s datasets. For the “Batch” dataset using the same platform, including GSE6575-GPL570 and GSE18123-GPL570, batch effects were removed using the ComBat function of the R package “SVA” (40). The 3D principal component analysis plots indicated that the processed data were more reliable after batch effect removal (**Figures 2A, B**). For “RRA” dataset using different platforms, including GSE18123-GPL6244 and GSE42133-GPL10558, the batch effect was corrected using the R package “RobustRankAggreg” (RRA) (41) (**Figure 2C**). As analyzed by “limma”, the final list of DEGs used for further analysis was generated as shown by the Venn diagram in **Figure 2D**. There were a total of 113 DEGs, of which 95 were consistently changing DEGs between these two datasets in the childhood group, including 57 co-upregulated and 38 co-downregulated genes (**Supplementary Table 2** and **Figure 2D**). For the two adult datasets, DEGs were extracted as the intersection between GSE26415 and GSE89594 as shown in **Supplementary Figure 1**. A total of 25 consistently changing DEGs were screened between the GSE26415 and GSE89594 adult datasets, including 17 co-upregulated and 8 co-downregulated genes (**Supplementary Table 3** and **Supplementary Figure 1A**).

### Functional and signaling pathway enrichment analysis in children and adults with ASD

**Figure 3:**
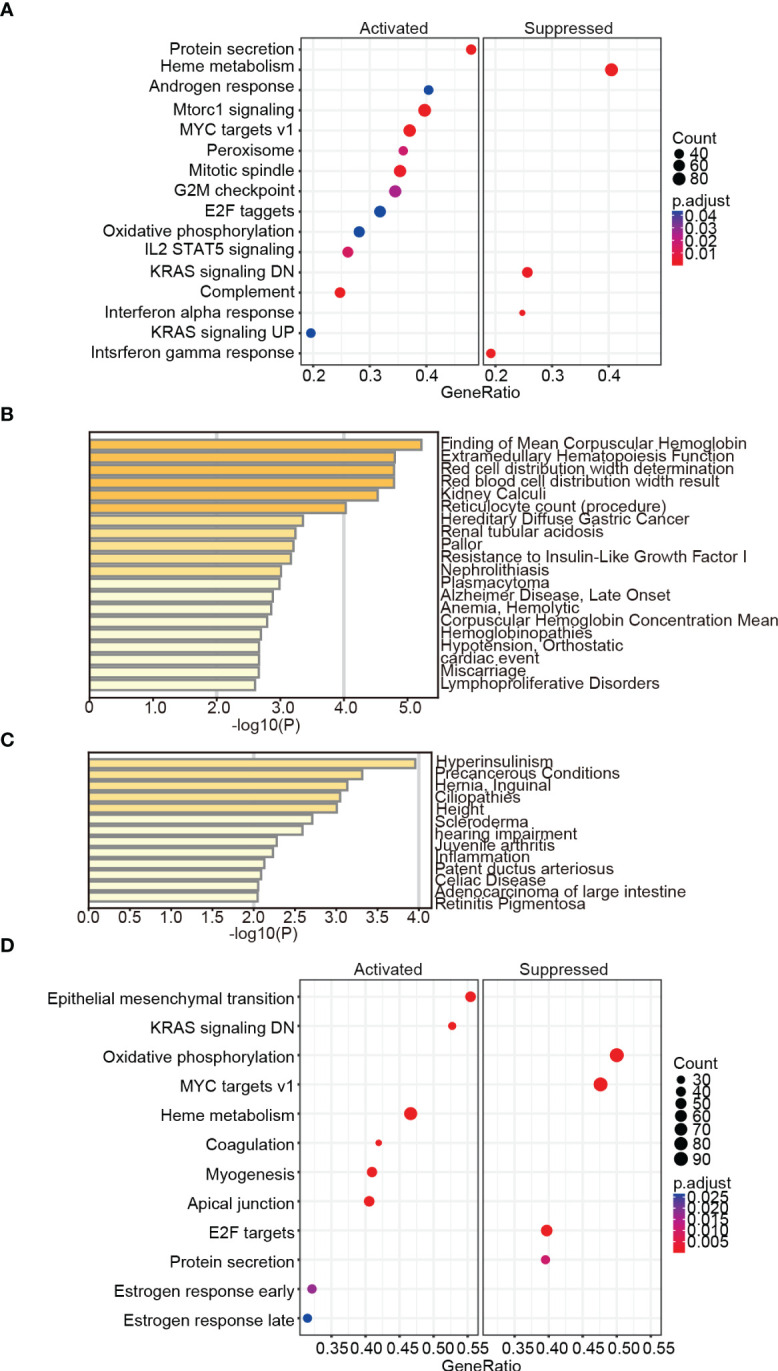
Gene enrichment analysis based on DisGeNET and GSEA. Summary of the enrichment analysis in DisGeNET in the children **(A)** and adult **(B)** groups. The GSEA results showed the enriched activated and suppressed pathways in children **(C)** and adults **(D)**.

To reveal the biological processes and signaling pathways behind the DEGs in children and adults, we performed a systematic analysis, including GO, KEGG, GSEA, and DisGeNET database prediction. For the childhood group, the 95 consistently changing DEGs were analyzed with Metascape and GSEA. In total, biological functions related to neuronal development (axon guidance, nervous system development, and Roundabout (ROBO) receptors), immunity (adaptive immune system, IFN-alpha/gamma response, complement, and IL2/Stat5 signaling), and cell development and metabolic processes (mTORC1 signaling, mitotic spindle formation, KRAS signaling, heme metabolism, and protein secretion) (**Table 1**; **Figure 3A**) were identified.

**Table 1:** The top four GO MCODE components identified in 95 consistently changing DEGs in the protein-protein interaction network.

GO	Description	Log10(*P*)	Gene sets
R-HSA-422475	Axon guidance	–6.7	*AP2A1*, *PRKCA*, *PSMC4*, *RPS3A*, *RPS26*, *YES1*, *IRS2*, *PSMF1*, *USP33*
R-HSA-9675108	Nervous system development	–6.6	*AP2A1*, *PRKCA*, *PSMC4*, *RPS3A*, *RPS26*, *YES1*, *IRS2*, *PSMF1*, *USP33*
R-HSA-376176	Signaling by ROBO receptors	–5.9	*PRKCA*, *PSMC4*, *RPS3A*, *RPS26*, *PSMF1*, *USP33*
R-HSA-1280218	Adaptive immune system	–5.5	*AP2A1*, *CD79A*, *CD81*, *KLRB1*, *PSMC4*, *YES1*, *PSMF1*, *UFL1*, *FBXO7*

Interestingly, the DisGeNET prediction suggested that the DEGs were mainly associated with erythrocyte-related parameters and disorders such as mean corpuscular hemoglobin (MCH), extramedullary hematopoiesis function, red cell distribution width (RDW), mean corpuscular hemoglobin concentration (MCHC), and anemia (**Figure 3B**; **Supplementary Table 4**). Indeed, these results were confirmed and validated when using peripheral blood samples collected from children with ASD. We observed significant differences between the ASD and TD groups for red blood cell count (RBC), hematocrit (HCT), MCH, coefficient of variation of RDW (RDW-CV), and MCHC (**Table 2**). Moreover, GO enrichment analysis of the DEGs suggested biological functions associated with erythrocyte differentiation (**Supplementary Figure 2**).

**Table 2:** The comparison of routine blood tests between TD and ASD groups in the clinical children’s samples.

	TD (n = 30)	ASD (n = 30)	*p*
WBC (10^9/L)	7.64 ± 1.55	8.30 ± 2.06	0.19
RBC (10^12/L)	4.40 ± 0.28	4.67 ± 0.41	<0.01
HGB (g/L)	126.33 ± 7.04	125.37 ± 9.80	0.77
HCT (%)	35.88 ± 1.82	37.63 ± 2.55	<0.01
MCV (fL)	81.68 ± 2.66	80.95 ± 5.85	0.82
MCH (pg)	28.77 ± 1.16	26.98 ± 2.09	<0.001
MCHC (g/L)	352.10 ± 6.46	333.23 ± 9.11	<0.001
RDW-CV (%)	12.30 ± 0.53	13.31 ± 0.96	<0.001
RDW-SD (fL)	37.77 ± 1.41	37.96 ± 2.26	0.71
PLT (10^9/L)	296.23 ± 74.43	316.53 ± 70.41	0.16
PCT (%)	0.27 ± 0.06	0.27 ± 0.07	0.82
MPV (fL)	9.17 ± 1.22	8.63 ± 1.24	0.06

WBC, white blood cell count; RBC, red blood cell count; HGB, hemoglobin; HCT, hematocrit; MCV, mean corpuscular volume; MCH, mean corpuscular hemoglobin; MCHC, mean corpuscular hemoglobin concentration; RDW-CV, coefficient of variation of red cell distribution width; RDW-SD, standard deviation of red cell distribution width; PLT, platelet count; PCT, plateletcrit; MPV, mean platelet volume.

It is noteworthy that when analyzing the functional enrichment of DEGs using the “RRA” and “Batch” method separately, we found that the B cell receptor (BCR) signaling pathway was significantly enriched in both (**Supplementary Figures 3A, B**). Similarly, when analyzing all 113 DEGs instead of the consistently changing 95 DEGs between the two methods using the Metascape database, we also found the BCR signaling pathway to be a significant pathway (**Supplementary Figure 3C**). In addition, several DEGs, including *PIR-B* (*LILRB1*), *IgA*, *CD81*, and *VAV*, were found to be involved in the BCR signaling pathway (data not shown).

For the adult group, Metascape analysis of the 25 consistently changing DEGs suggested that deubiquitination, regulation of hormone levels, cell morphogenesis involved in neuron differentiation, and positive regulation of catabolic process were the most enriched terms (**Supplementary Figure 1B**). GENEMANIA analysis showed that the TGF-β/SMAD signaling pathway was a significantly altered signaling pathway (**Supplementary Figure 1C**), and some metabolic disorders and immune disease-related processes were significantly enriched according to the DisGeNET analysis, such as hyperinsulinism, scleroderma, juvenile arthritis, inflammation, and celiac disease (**Figure 3C**). In addition, GSEA analysis showed that oxidative phosphorylation, MYC targets v1, E2F targets, and protein secretion were suppressed, while heme metabolism and KRAS signaling DN were activated, all of which were the opposite of what was seen in the childhood group. It is also noteworthy that estrogen response was among the biological processes enriched in the adult ASD datasets compared to children (**Figure 3D**).

Overall, the biological process analysis using DEGs showed that immune function, neuronal development, and metabolic disorders were common in both children and adults with ASD. However, there were obvious differences between the two groups. For example, erythrocyte differentiation was involved in children with ASD, while estrogen response affected the course of adults with ASD.

### Immune cell landscape in peripheral blood samples of children with ASD

The analysis above clearly showed that immune function is among the most significantly affected biological functions in both children and adults with ASD. In addition, immune responses and inflammation have been suggested to play a crucial role in childhood ASD progression, so we conducted a comprehensive analysis of the immune cell profiles in the selected datasets. The “Batch” dataset for children (101 ASD children and 45 TD children) and the GSE26415 dataset for adults (21 ASD adults and 21 TD adults) were used because of the high quality of their sequencing data and the availability of the CIBERSORT algorithm.

**Figure 4:**
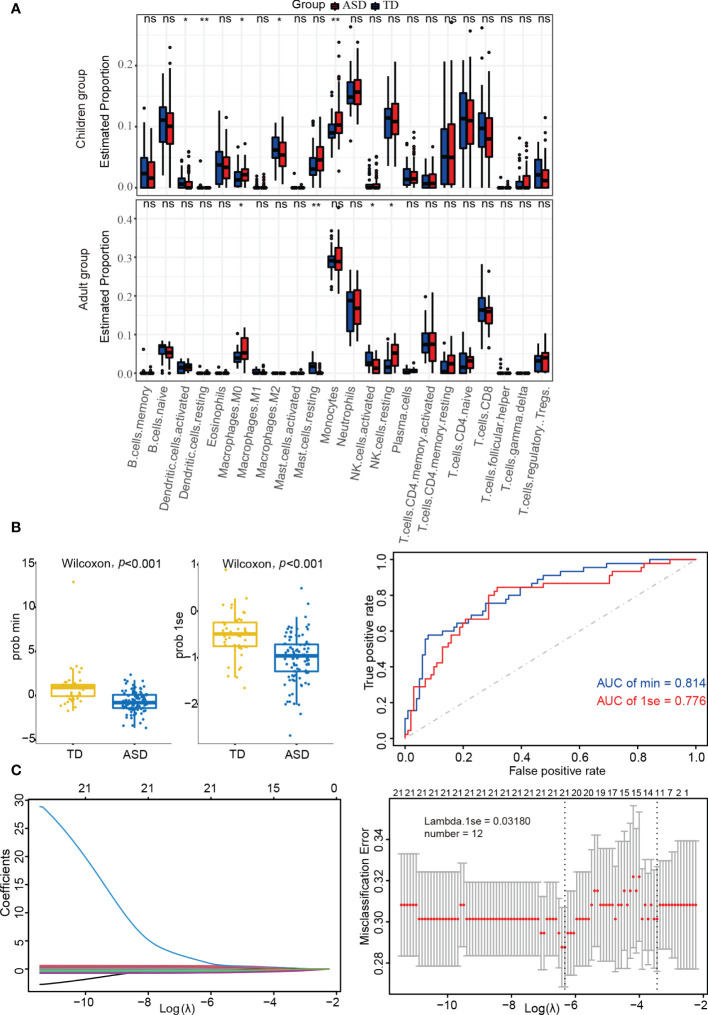
Identifying the significantly different infiltrations of immune cells. **(A)** Kruskal test analysis in children and adults. **(B)** LASSO regression analysis in children’s datasets. **(C)** Box-plots and ROC curves for assessing classifier performance in the LASSO regression model. ns: not significant; *p < 0.05; **p < 0.01.

Using the CIBERSORT algorithm, 22 immune cell types were identified in each sample from each group (**Supplementary Figure 4**). Among these, naive B cells, monocytes, neutrophils, resting NK cells, naive CD4^+^ T cells, and CD8^+^ T cells were the main immune cells in children, while monocytes, neutrophils, memory-activated CD4^+^ T cells, and CD8^+^ T cells were similarly enriched in adults. In addition, there were significant differences between the ASD and TD groups regarding resting/activated dendritic cells, M0/M2 macrophages, and monocytes in children and M0 macrophages, resting mast cells, and resting/activated NK cells in adults (**Figure 4A**).

LASSO regression analysis can more accurately identify significant differences in immune cells. As shown in **Supplementary Table 5** and **Figure 4B**, the LASSO regression model was found to have good classifier performance according to Wilcoxon’s test and area under the ROC curve (AUC) analysis. When further analyzing the data in children with ASD, 12 types of immune cells were extracted (**Figure 4C**). Among these, four cell types (monocytes, M2 macrophages, and resting/activated dendritic cells) from two algorithms (Kruskal.test and LASSO logistic regression) were identified.

### Monocyte phenotype validation and confirmation using peripheral blood in children with ASD

**Figure 5:**
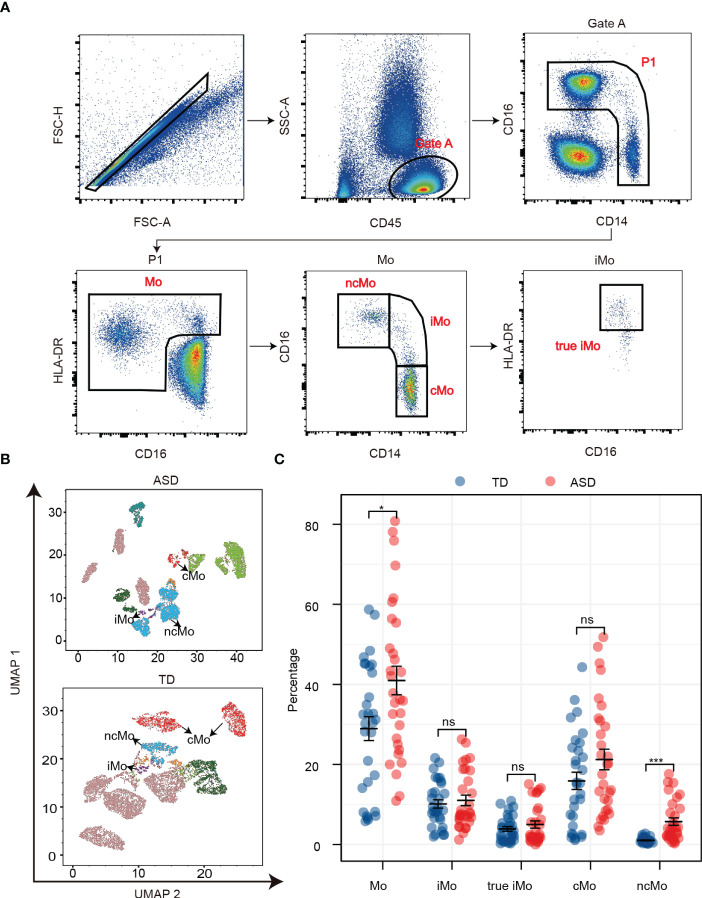
The expression of the monocyte phenotype. **(A)** Monocytes were typed using flow cytometry. **(B)** UMAP for monocytes and monocyte subtypes in children with ASD and TDs. **(C)** The percentage of monocytes and subtypes in dot plots. *p < 0.05; ***p < 0.001. Mo, monocyte; iMo, intermediate monocyte; cMo, classical monocyte; ncMo, nonclassical monocyte; ns, not significant.

It has been suggested that monocytes play a major role in ASD development in children (42). To validate the results obtained from the integration analysis above, monocyte phenotypes were further identified and confirmed using peripheral blood samples. We collected a total of 60 peripheral whole blood samples, including 30 ASD and 30 age and sex-matched TD children, and performed flow cytometry analysis (**Figure 5A**). In the UMAP analysis, each of the monocyte subtype populations, including intermediate monocytes, classical monocytes, and nonclassical monocytes (ncMos), were shown as distinct cell clusters (**Figure 5B**). The flow cytometry analysis showed that among all analyzed monocyte subtypes, there were significant differences in total monocytes (*p* < 0.05) and ncMos (*p* < 0.001) (**Figure 5C**).

### Screening and validation of hub genes as predictors in children with ASD

Hub genes usually refer to genes with a connectivity degree greater than 10 in the genetic interaction network, and these play important roles in biological systems. Here, we used the “Batch” and “RRA” datasets as the training dataset and GSE111175 as the validation dataset for children with ASD. Notably, due to the integration and consistency of the data in the “Batch” and “RRA” datasets, we used the expression data from the “Batch” dataset to represent the expression levels of the hub genes in the training dataset.

**Figure 6:**
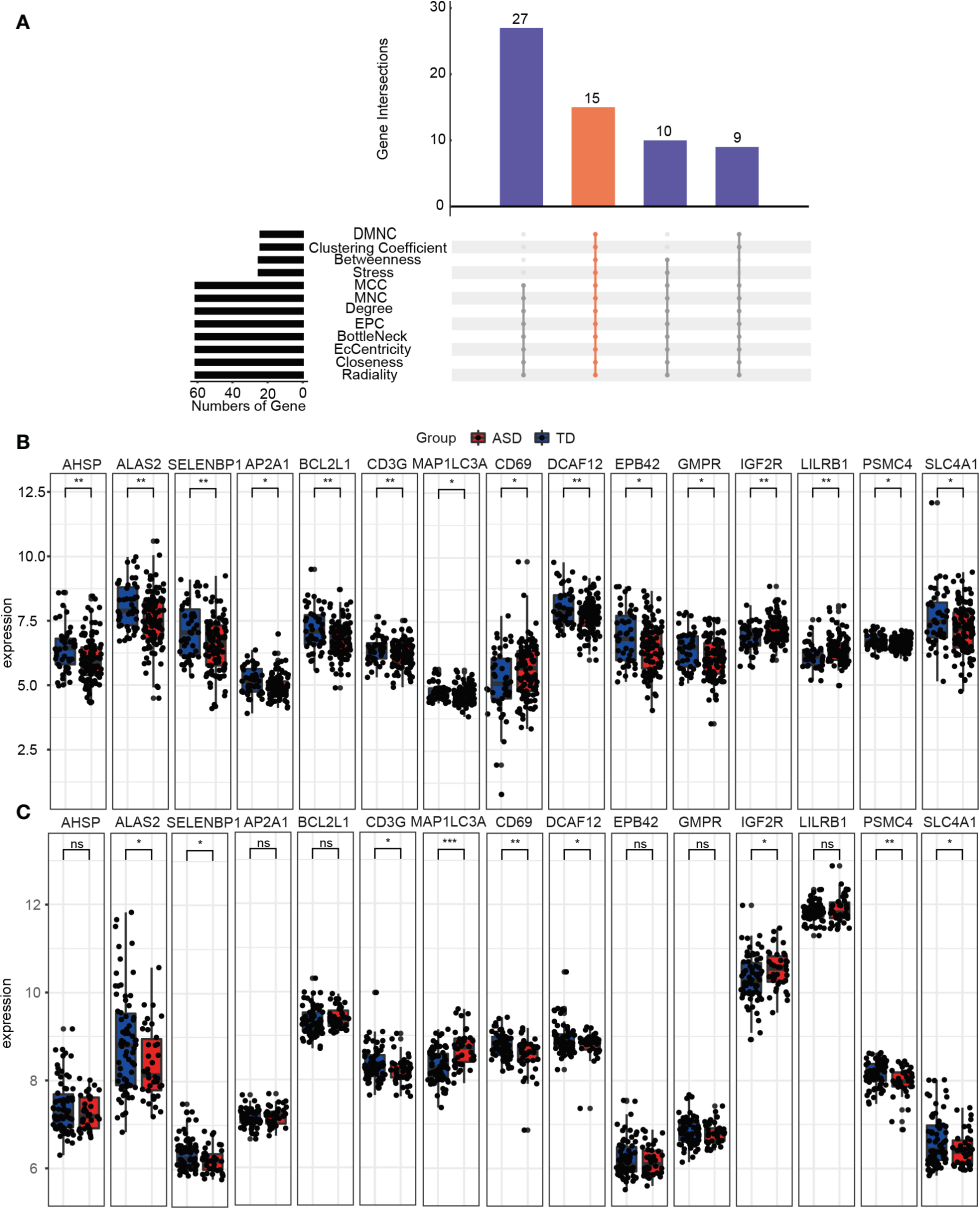
Identifying the hub genes. **(A)** The 12 algorithms used to screen for hub genes in the R package “UpSet”. Expression levels of 15 hub genes in the **(B)** training and **(C)** validation groups. ns, not significant; *p < 0.05; **p < 0.01; ***p < 0.001.

The PPI network used to identify the hub genes was obtained when 95 consistently changing DEGs in children with ASD were analyzed by STRING (**Supplementary Figure 5**). Using the CytoHubba function, there were a total of 15 hub genes (**Figure 6A**), including *AHSP*, *ALAS2*, *SELENBP1*, *AP2A1*, *BCL2L1*, *CD3G*, *MAP1LC3A*, *CD69*, *DCAF12*, *EPB42*, *GMPR*, *IGF2R*, *LILRB1*, *PSMC4*, and *SLC4A1*, and the expression levels of the hub genes were determined in the training (**Figure 6B**) and validation (**Figure 6C**) datasets in children with ASD and TD. Clearly, the validation dataset confirmed only *ALAS2*, *SELENBP1*, *CD3G*, *MAP1LC3A*, *CD69*, *DCAF12*, *IGF2R*, *PSMC4*, and *SLC4A1* as significantly differently expressed hub genes between children with ASD and TD and not the genes *AHSP*, *AP2A1*, *BCL2L1*, *EPB42*, *GMPR*, and *LILRB*.

**Figure 7:**
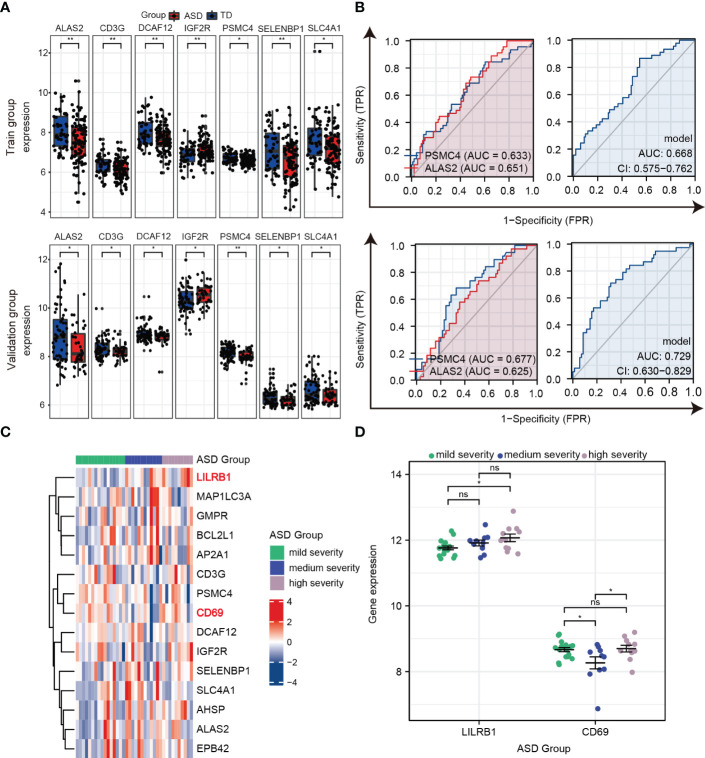
Verifying the predictors from hub genes in the training and validation groups. **(A)** Seven hub genes with consistent changes in expression levels in the training group (top) and validation group (bottom). **(B)** ROC curve and logistic regression model to assess the predictive accuracy of *PSMC4* and *ALAS2* in the training group (top) and validation group (bottom). **(C)** Heat maps of the expression of 15 hub genes in the three groups according to ASD severity. **(D)** Prognostic values for *LILRB1* and *CD69* in dot plots. ns, not significant; *p < 0.05; **p < 0.01.

Furthermore, as shown in **Figure 7A**, *ALAS2*, *CD3G*, *DCAF12*, *IGF2R*, *PSMC4*, *SELENBP1*, and *SLC4A1* were significantly and consistently altered hub genes in the training and validation datasets, which suggested that these genes were critical to ASD and might be used as predictors for ASD in children. An AUC more than 0.60 was considered to be relatively good predictive accuracy. The AUC values of *PSMC4* and *ALAS2* were 0.633 (95% CI 0.534–0.732) and 0.651 (95% CI 0.559–0.744) in the training group and 0.677 (95% CI 0.574–0.779) and 0.625 (95% CI 0.517–0.733) in the validation group, respectively. In the logistic regression model the combined AUC values of *PSMC4* and *ALAS2* reached 0.668 (95% CI 0.575–0.762) and 0.729 (95% CI 0.630–0.829), respectively, in both datasets (**Table 3**; **Figure 7B**). In addition, we also undertook diagnostic performance analyses of the remaining five possible marker genes, including single and multiple gene combination diagnostic analyses. However, we did not find any additional predictors in the two datasets when considering higher diagnostic efficiency and lower cost. Therefore, *PSMC4* and *ALAS2* might act as predictors with moderate strength (43).

**Table 3:** Receiver operative characteristic curves of *PSMC4*, *ALAS2*, and Model (*PSMC4*+*ALAS2*) in the training and validation group.

		Level		Sensitivity		Specificity		95% CI		AUC		
		TD	ASD									
Training group	*PSMC4*	6.72 ± 0.28	6.60 ± 0.23	0.84		0.40		0.534-0.732		0.633	
	*ALAS2*	8.09 ± 0.88	7.53 ± 1.07	0.73		0.52		0.559-0.744		0.651	
	Model	-0.59 ± 0.66	-1.02 ± 0.65	0.87		0.46		0.575-0.762		0.668	
Validation group	*PSMC4*	8.16 ± 0.29	7.96 ± 0.33	0.68		0.67		0.574-0.779		0.677	
	*ALAS2*	8.86 ± 1.15	8.37 ± 0.81	0.74		0.49		0.517-0.733		0.625	
	Model	-0.96 ± 0.85	-0.26 ± 0.88	0.71		0.69		0.630-0.829		0.729	

To further estimate the sensitivity of using these hub genes as indicators for ASD severity, we extracted the Autism Diagnostic Observation Schedule Social Affect (ADOS-SA) deficit scores that were positively correlated with ASD severity from the GSE111175 dataset. The children with ASD were divided into the following three groups according to severity: mild (5 to 11), medium (12 to 15), and high (16 to 21). The heatmap showed the expression of 15 hub genes in the three groups (**Figure 7C**), and we used scatter plots to show gene expression patterns among the three groups to make it more intuitively understandable. In particular, *LIlRB1* could distinguish between high and mild severity ASD (*p* < 0.05), and *CD69* had relatively lower expression in the medium severity group compared to the mild and high severity groups (*p* < 0.05) (**Figure 7D**). These results indicate that *LIlRB1* and *CD69* can be used as indicators for ASD severity in children.

### Correlation between hub genes and peripheral immune cell profiles in children with ASD

**Figure 8:**
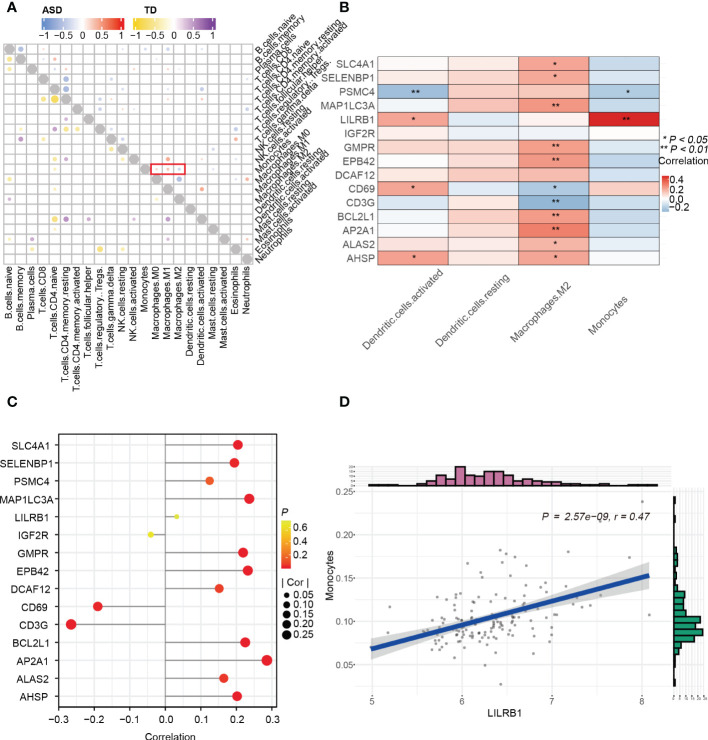
Correlation between immune cells and genes. **(A)** Correlation between 15 hub genes and 4 significantly differential immune cells. **(B)** Lollipop diagram of the correlation between 15 hub genes and M2 Macrophages with |r| > 0.20, *p* < 0.05. **(C)** Correlation among immune cells in the child ASD and TD groups. **(D)** Scatter plot of significantly related LIlRB1 and monocytes with r > 0.40 and *p* < 0.001. *p < 0.05; **p < 0.01.

To investigate the correlations between hub genes and immune cell profiles in children with ASD, we performed Spearman correlation analysis using the “Batch” dataset. First, we explored the correlation between immune cells in children with ASD and TD, and we found that monocytes were positively associated with M0/M1 macrophages but negatively related to M2 macrophages in children with ASD, whereas M0 macrophages were negatively related to M2 macrophages in TD children (**Figure 8A**). Second, the correlation between 15 hub genes and 4 significantly differentially expressed immune cells (**Figure 8B**) showed that activated dendritic cells were positively correlated with *AHSP*, *LILRB1*, and *CD69* (r > 0.20, *p* < 0.05) but negatively correlated with *PSMC4* (r < –0.20, *p* < 0.05). In addition, M2 macrophages showed a positive correlation with *AP2A1*, *MAP1LC3A*, *EPB42*, *BCL2L1*, *GMPR*, *SLC4A1*, and *AHSP* (r > 0.20, *p* < 0.05) and a negative correlation with *CD3G* (r < –0.20, *p* < 0.05) in children with ASD (**Figure 8C**). Lastly, the graphs of the linear correlation indicated that monocytes were significantly and positively related to *LIlRB1* (r > 0.40, *p* < 0.001) (**Figure 8D**).

### Construction of the miRNA-hub gene network and ASD-related agents’ prediction in children with ASD

To gain further insight into the regulatory mechanisms and to identify potential agents affecting ASD in children, we next tried to identify the miRNAs and agents that influence identified 15 hub genes.

**Figure 9:**
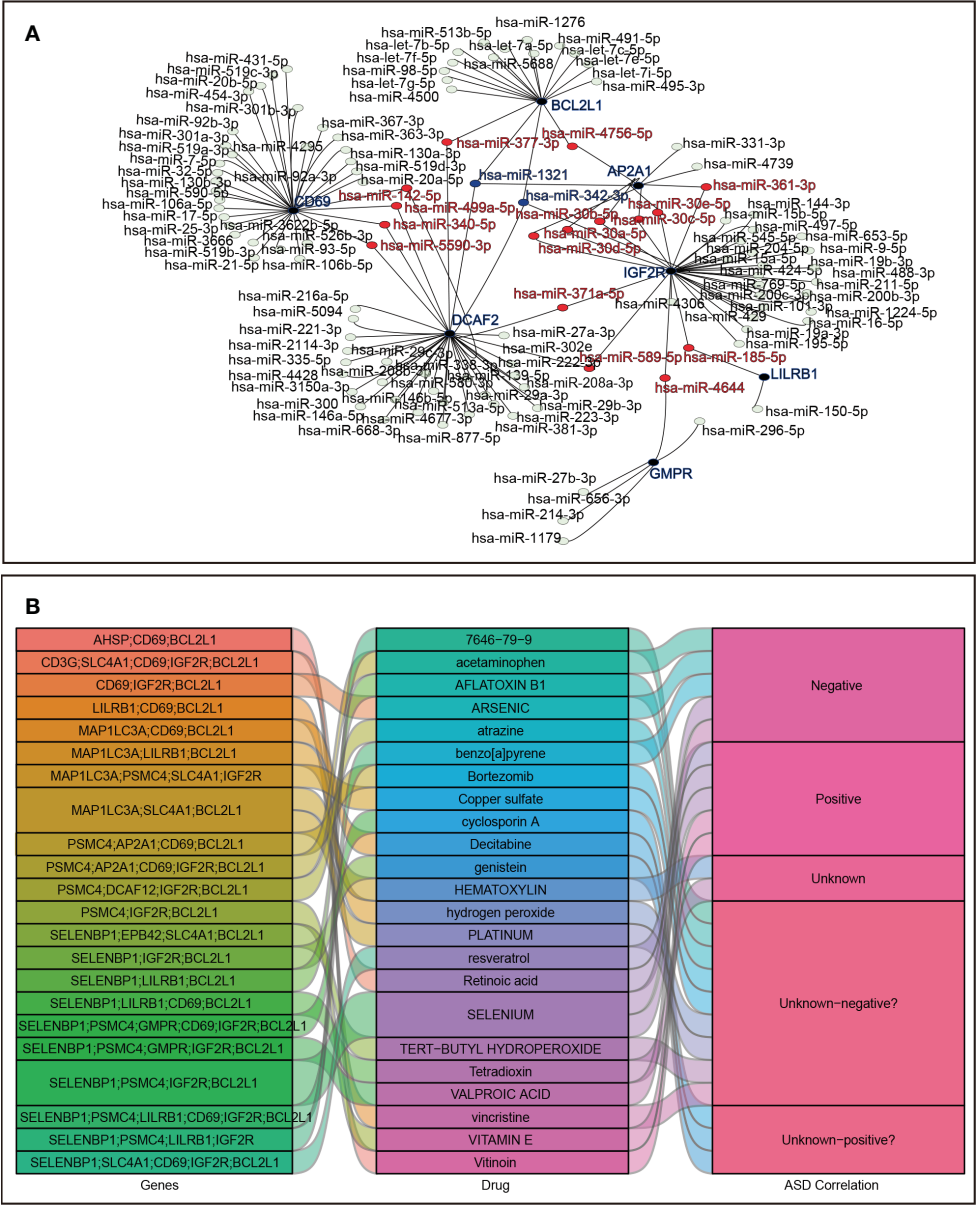
Prediction in miRNA-mRNA and agents-genes. **(A)** miRNA-mRNA network. **(B)** Sankey diagram for agents–hub genes.

A total of 118 candidate miRNAs were predicted to target the 7 identified hub genes. There were 10 miRNAs targeting *AP2A1*, 18 miRNAs targeting *BCL2L1*, 33 miRNAs targeting *CD69*, 37 miRNAs targeting *DCAF2*, 5 miRNAs targeting *GMPR*, 33 miRNAs targeting *IGF2R*, and 2 miRNAs targeting *LIlRB1* (**Figure 9A**). We then summarized those miRNAs that regulated more than one hub gene in **Table 4**. Of these, hsa-miR-342-3p and hsa-miR-1321 interacted most intensively with robust hub genes (target hub genes = 3).

**Table 4:** The candidate miRNAs that regulate multiple target genes in the miRNA-hub genes network.

miRNAs	Target genes	miRNAs	Target genes
hsa-miR-30a-5p, hsa-miR-30e-5p, hsa-miR-30d-5p, hsa-miR-30c-5p, hsa-miR-361-3p, hsa-miR-30b-5p	*AP2A1*, *IGF2R*	hsa-miR-142-5p, hsa-miR-5590-3p, hsa-miR-340-5p, hsa-miR-499a-5p	*DCAF2*, *CD69*
hsa-miR-589-5p, hsa-miR-371a-5p	*DCAF2*, *IGF2R*	hsa-miR-4644	*GMPR*, *IGF2R*
hsa-miR-4756-5p	*BCL2L1*, *AP2A1*	hsa-miR-342-3p	*BCL2L1*, *DCAF2*, *IGF2R*
hsa-miR-377-3p	*BCL2L1*, *DCAF2*	hsa-miR-1321	*BCL2L1*, *DCAF2*, *AP2A1*
hsa-miR-185-5p	*LIlRB1*, *IGF2R*		

In addition, using the DSigDB database, the 23 agents affecting ASD were shown in a Sankey diagram with agents–hub genes ≥ 3, and the agents-disease associations were also displayed, including positive, negative, and unknown correlations with ASD status (**Figure 9B**).

## Discussion

This study identified seven ASD-related hub genes (*PSMC4*, *CD3G*, *IGF2R*, *DCAF12*, *SELENBP1*, *ALAS2*, *SLC4A1*) and found that *PSMC4* and *ALAS2* had good diagnostic efficacy. In addition, *LIlRB1* and *CD69* had the ability to diagnose mild, medium, and high severity ASD. Among these, *CD3G* encodes the CD3-gamma polypeptide, which plays an important role in autoimmunity. It has been reported that CD3γ-deficient patients present with Treg phenotypic and functional defects (44); however, no study has been reported on the relationship between *CD3G* and ASD. Here, we found that the expression of *CD3G* was lower in children with ASD, which shows that there were Treg cell abnormalities in these children (45). Both Treg cells and M2 macrophages can suppress overly aggressive immune responses, and thus loss of Treg cell function may require more M2 macrophages to restore immunosuppressive function, which could be the reason that *CD3G* had a negative correlation with M2 macrophages.

*LILRB1* and *CD69* are essential genes in immune regulation through T cells and play roles in the development of autoimmune responses (46–49). The most intriguing result in our study was that *LILRB1* was tightly related to ASD. *LILRB1* is expressed mainly in myeloid lineage cells like monocytes and dendritic cells, and because *LILRB1* expression increases with progressive ASD severity the circulating monocyte numbers increase progressively with advancing disease, which is similar to previous reports (50). These results suggest that LIlRB1 plays a crucial role in ASD processes, and in-depth explorations will provide us with a better understanding of the mechanisms of autism. CD69, the early T cell activation marker, can promote the proliferation and activation of T cells, and dendritic cells act as professional antigen-presenting cells to initiate T cell-mediated immune responses, which could explain why *CD69* was positively related with activated dendritic cells in our study.

SELENBP1, as its name suggests, combines with selenium and participates in various intracellular selenium transport mechanisms (51). Hence, deficiency of SELENBP1 causes deficiency of selenium, leading to certain neurologic diseases such as recent-onset schizophrenia (52) and autism (53, 54). Consistent with other studies, we also observed that SELENBP1 was lower in ASD children than in TD children, which indicates that low selenium levels is a risk indicator for autism. This finding provides further evidence for the addition of selenium supplements to ASD treatments.

IGF2R, the receptor that binds to insulin-like growth factor 2 (IGF2), is important for fetal growth and development (55). IGF2 *via* IGF2R reverses the abnormal activity of the AMPK-mTOR-S6K pathway and rescues active translation at synapses in autism-like phenotypes in mice (56), and bidirectional regulation of IGF2 in autoimmune diseases has been suggested to be related to the differential activation of IGF1R and IGF2R, which regulates both the anti- and pro-inflammatory effects of macrophages (57). Thus IGF2R plays a key role in neuronal development, and its increased expression in cases of ASD implies that there are different degrees and types of activation of macrophages in ASD. As we expected, M0 macrophages levels were increased in ASD patients, which indicates that there are abnormalities in immunologic homeostasis. In addition, the decreased numbers of M2 macrophages and increased numbers of monocytes suggest that children with ASD are in a relatively pro-inflammatory state.

DCAF12 and PSMC4 are both involved in the degradation of ubiquitinated proteins (58, 59), but there have been no studies showing a relation between these two genes and autism. Here we observed that *DCAF12* and *PSMC4* in the ASD group had reduced expression compared to TD group. This suggests that there might be a problem with the degradation of ubiquitinated proteins in children with autism. Therefore, we further explored whether there might be another pathway for protein degradation, such as autophagy. Interestingly, MAP1LC3A, also known as microtubule-associated protein light chain 3 (LC3), is lipidated to form LC3-II and is generally considered to be a good indicator of macroautophagy (60). We found that *MAP1LC3A* was a DEG in both the training and validation groups. Although the trends were opposite in these two groups, we cannot exclude that autophagy takes part in the development of autism. In addition to macroautophagy’s role in protein degradation, local protein is also cleared by dendritic cells. We found that *PSMC4* was negatively correlated with activated dendritic cells, which suggests that more activated dendritic cells might prevent the accumulation of proteins due to the reduced degradation caused by decreased PSMC4.

In the DEG analysis, erythrocyte differentiation, erythrocyte-related disorders, and heme metabolism were enriched in children with ASD, and our clinical data from routine blood tests validated the significant changes in erythrocyte-related parameters (RBC, HCT, MCH, RDW-CV, and MCHC) in ASD patients. Erythrocyte parameters can reflect iron status (61), and thus the results presented above imply that abnormalities in iron metabolism affect the course of ASD, as reported in previous studies (62, 63). *ALAS2* (64, 65), *SLC4A1* (66), *AHSP* (67), *EPB42* (68), and *GMPR* (69) were identified as ASD hub genes, and these are associated with erythrocytes, especially with regard to changes in hemoglobin, which are closely related to iron metabolism. A review article summarized the evidence showing that iron content regulates macrophage polarization (70). Our study found that *SLC4A1* had a positive correlation with M2 macrophages, which suggests that decreased SLC4A1 affects erythrocyte formation and may cause an imbalance in heme-iron metabolism in macrophages and may further result in a decrease in the production of anti-inflammatory M2 macrophages. In summary, our data provide genetic and cellular evidence supporting the correlation between iron metabolism abnormalities and ASD, and further studies are needed to validate these findings. Compared to children, hormone levels and early/late estrogen response were enriched in adults with ASD. Although recent research reported that prenatal estrogens contribute to the risk of autism (71), there is no study on the link between adults with ASD and estrogen. A previous study showed that estrogen is indispensable for immune robustness and for neural functions (72), so abnormal activation of estrogen may contribute to immune and neurological abnormalities in adults with ASD.

In the GSEA analysis in adults with ASD, the MYC targets v1, E2F targets, protein secretion, heme metabolism, and KRAS signaling DN showed opposite trends in inhibition or activation compared to children with ASD. These age-related differences in ASD may indicate distinct disease characteristics between children and adults, and this suggests the need for different approaches to raising awareness and adjusting treatment for ASD patients in different age groups.

In terms of the immune cell landscape, decreased activated NK cells and increased resting NK cells suggest that reduced NK cell number and activity may result in immune disorders in adults with ASD. In children with ASD, we postulate that ncMos are abnormal. A recent article showed that ncMos regulate autoimmune and inflammatory diseases (73) and can directly regulate the adaptive immune response by regulating the activity of specific subpopulations of other immune cells. Hence, the significantly elevated ncMo levels indicate that ASD is a class of immune-mediated disorders, especially disorders in the adaptive immune system. Also, identifying ncMos that exhibit specific pathogenic roles is important for the development of targeted autism therapies.

We predicted that *BCL2L1*, *DCAF2*, and *IGF2R* are the target genes of miR-342-3p and that *BCL2L1*, *DCAF2*, and *AP2A1* are the target genes of miR-1321, which suggested miRNAs’ therapeutic value in treating ASD. Previous studies showed that miR-342-3p controls macrophage survival by targeting *BCL2L1* (74) and that miR-342 is up-regulated in lymphoblastoid cell lines in autism cases (75). In addition, hsa-miR-1321 has been suggested to play a role in the development of cancer (76, 77). However, there are no data reporting these miRNA-gene regulatory axes in peripheral blood samples from children with ASD. Because the identified hub genes are tightly linked to the initiation and progression of ASD, the targeted therapies using miR-342-3p and miR-1321 might be a promising novel treatment modality for autism.

We further found identified agents-gene axes using the DSigDB database. The existing studies have shown different types of agents affecting ASD, including those that improve ASD (resveratrol, vitinoin, vitamin E, and retinoic acid) (78–82) and those that exacerbate ASD (valproic acid, arsenic, benzo[a]pyrene, and acetaminophen) (83–86). Moreover, there are ASD-related agents with unknown risks, including reagents with neurotoxicity and immunotoxicity (platinum, bortezomib, tert-butyl hydroperoxide, vincristine, atrazine, 7646-79-9, hydrogen peroxide, aflatoxin B1, and copper sulfate) (87–95) or agents providing neuroprotection and immune regulation (genistein, cyclosporin A, and decitabine) (96–98) and agents that require exploration in terms of immune and neurological function (hematoxylin and tetradioxin). Interestingly, selenium has a bidirectional modulatory effect on autism, and supplementation with selenium can improve autism-like behaviors in animal models (99), while prenatal exposure to high levels of selenium may affect childhood neurodevelopment and induce ASD (100). Overall, these agents play distinct roles in the ASD process, which suggests that agents should be tailored to the biological functions of the hub genes to be targeted.

There are some limitations in our study. First, the datasets were from different GEO datasets, and there was not enough corresponding clinical information for assessment and prediction. Second, we focused on ASD in children due to the lack of more readily available adult datasets and samples, and this hindered us from exploring difference between children and adults in a comprehensive and objective manner. Third, we analyzed ASD in children and adults as groups with no details regarding specific ages, which may mask the greater and/or earlier age-related changes. Fourth, we used bioinformatics methods to screen hub genes and miRNA, and experimental verification of such interactions is lacking. Fifth, the clinical sample size was small, which may not support rigorous statistical analysis.

## Conclusion

In summary, our study identifies four potential blood predictors (*PSMC4*, *ALAS2*, *LIlRB1*, and *CD69*), four dysregulated immune cell types (monocytes, M2 macrophages, and resting and activated dendritic cells), two miRNAs (miR-342-3p and miR-1321) and 23 potential agents affecting ASD. The gene expression profiles are age-related in ASD, and we show for the first time that ncMos are upregulated in children with ASD. This study enriches our knowledge of the molecular mechanisms of ASD and promotes the development of early diagnosis and treatment strategies for childhood ASD.

## Data availability statement

Publicly available datasets were analyzed in this study. This data can be found here: GEO database (http://www.ncbi.nlm.nih.gov/geo) GSE6575, GSE18123, GSE42133, GSE111175, GSE26415, and GSE89594.

## Ethics statement

The studies involving human participants were reviewed and approved by The Medical Ethics Committee of the 3rd Affiliated Hospital of Zhengzhou University. Written informed consent to participate in this study was provided by the participants’ legal guardian/next of kin. Written informed consent was obtained from the individual(s), and minor(s)’ legal guardian/next of kin, for the publication of any potentially identifiable images or data included in this article.

## Author contributions

HL analyzed the data and wrote the manuscript. YX designed the study and analyzed the data. WL provided the clinical data of ASD patients. LZ conducted the validation experiments. XZ, BL, YC, and XW ensured the accuracy and integrity of the results. CZ devised and supervised the study. All authors contributed to the article and approved the submitted version.
