# Supplementary material for: Novel insights into the immune cell landscape and gene signatures in autism spectrum disorder by bioinformatics and clinical analysis
Source: Front Immunol. 2023 Jan 25;13:1082950. doi: 10.3389/fimmu.2022.1082950 (PMC9905846; doi:10.3389/fimmu.2022.1082950)
Supplement: Supplementary file 1 [file DataSheet_1.docx]

Supplementary Material

# Supplementary Data

**Supplementary Figures**

**Supplementary Figure 1.** The results of functional enrichment analyses of the 25 DEGs in adult ASD groups. (A) The Venn diagram of the 25 consistently changing DEGs between the two datasets. (B) Bar graph of enriched terms across 25 DEGs. (C) The network analysis from GENEMANIA.

**Supplementary Figure 2.** Network analysis of 95 consistently changing DEGs. (A) Network of enriched terms for 95 DEGs. (B) Bar graph of enriched terms across DEGs.

**Supplementary Figure 3.** The GO and KEGG analyses results of DEGs determined by different methods. (A) “Batch” method. (B) “RRA” method. (C) The intersection of “Batch” and “RRA” method.

**Supplementary Figure 4.** The proportion of 22 types of immune cells in each sample from the child and adult groups. (A) The histogram of the composition of immune cells. (B) The box-plots of the composition of immune cells.

**Supplementary Figure 5.** PPI network analysis results of 95 consistently changing DEGs by STRING.

**Supplementary Tables**

**Supplementary Table 1.** Basic information of selected ASD datasets.

**Supplementary Table 2.** 95 consistently changing DEGs between RRA and Batch.

**Supplementary Table 3.** 25 consistently changing DEGs between GSE26415 and GSE89594.

**Supplementary Table 4.** Summary of enrichment analysis in erythrocyte-related parameters and disorders in DisGeNET for the 95 consistently changing DEGs.

**Supplementary Table 5.** Receiver operative characteristic curves of prob_min and prob_1se in the LASSO regression model.

# Supplementary Figures and Tables

## Supplementary Figures


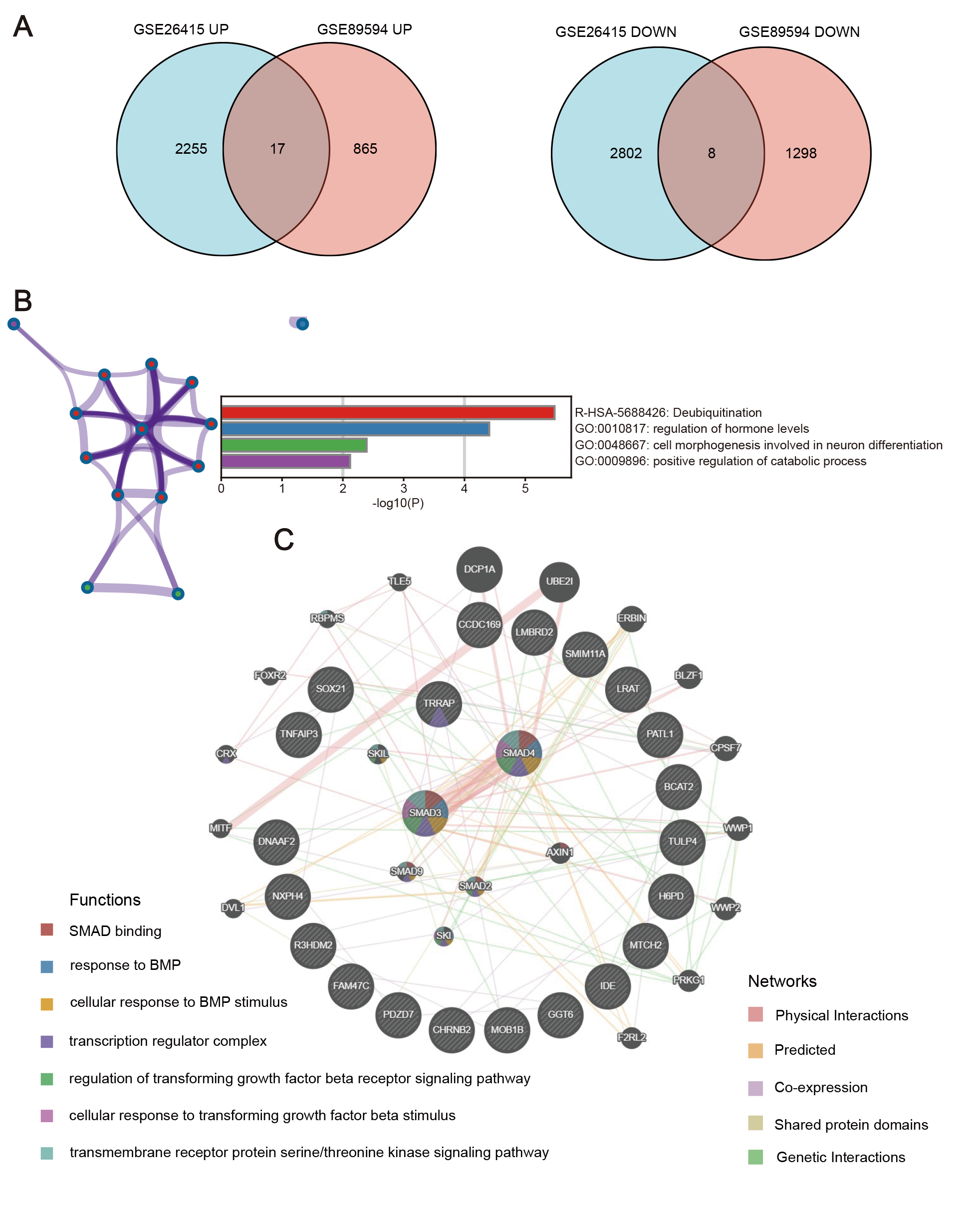


**Supplementary Figure 1.** The results of functional enrichment analyses of the 25 DEGs in adult ASD groups. (A) The Venn diagram of the 25 consistently changing DEGs between the two datasets. (B) Bar graph of enriched terms across 25 DEGs. (C) The network analysis from GENEMANIA.


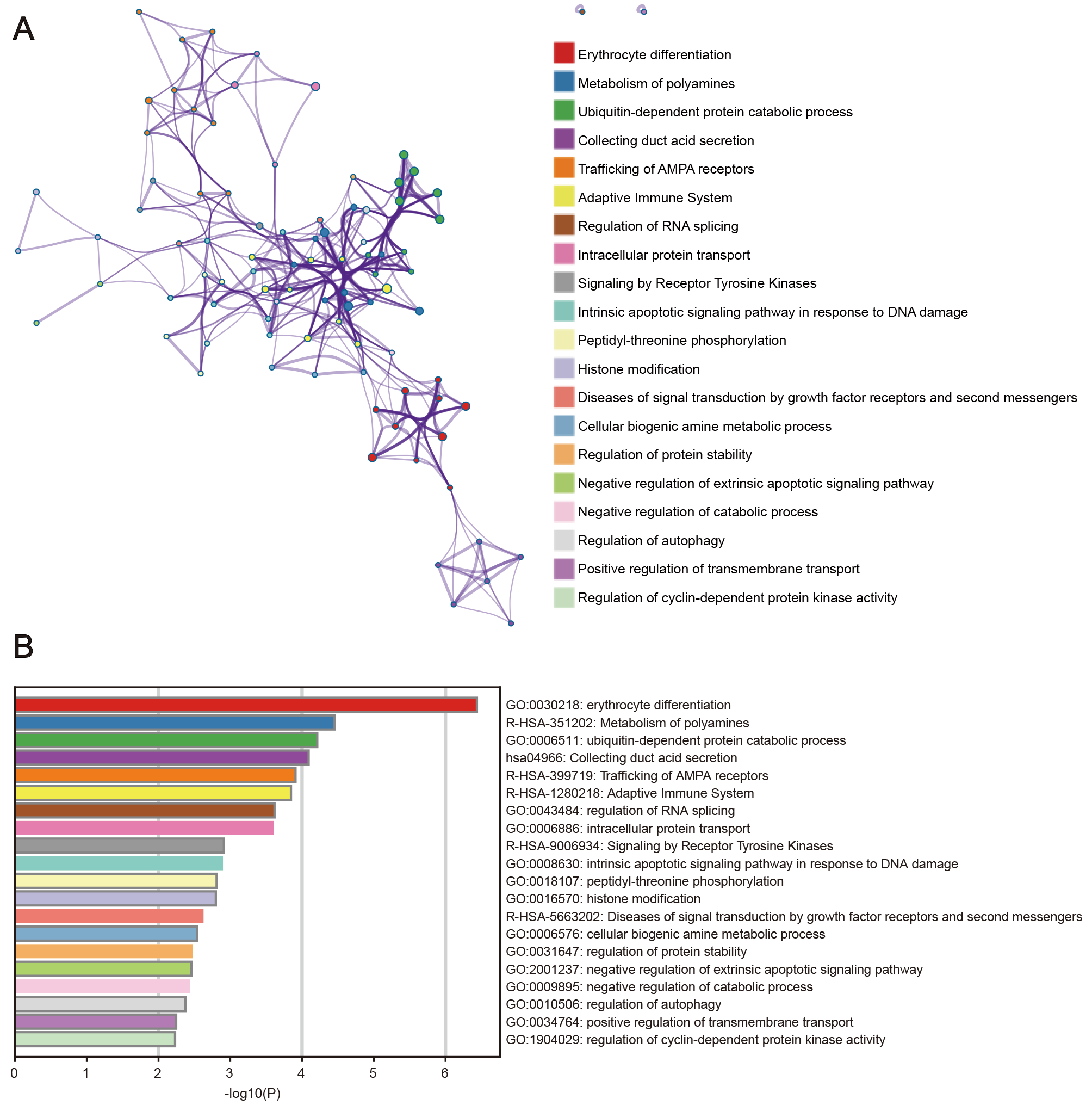


**Supplementary Figure 2.** Network analysis of 95 consistently changing DEGs. (A) Network of enriched terms for 95 DEGs. (B) Bar graph of enriched terms across DEGs.


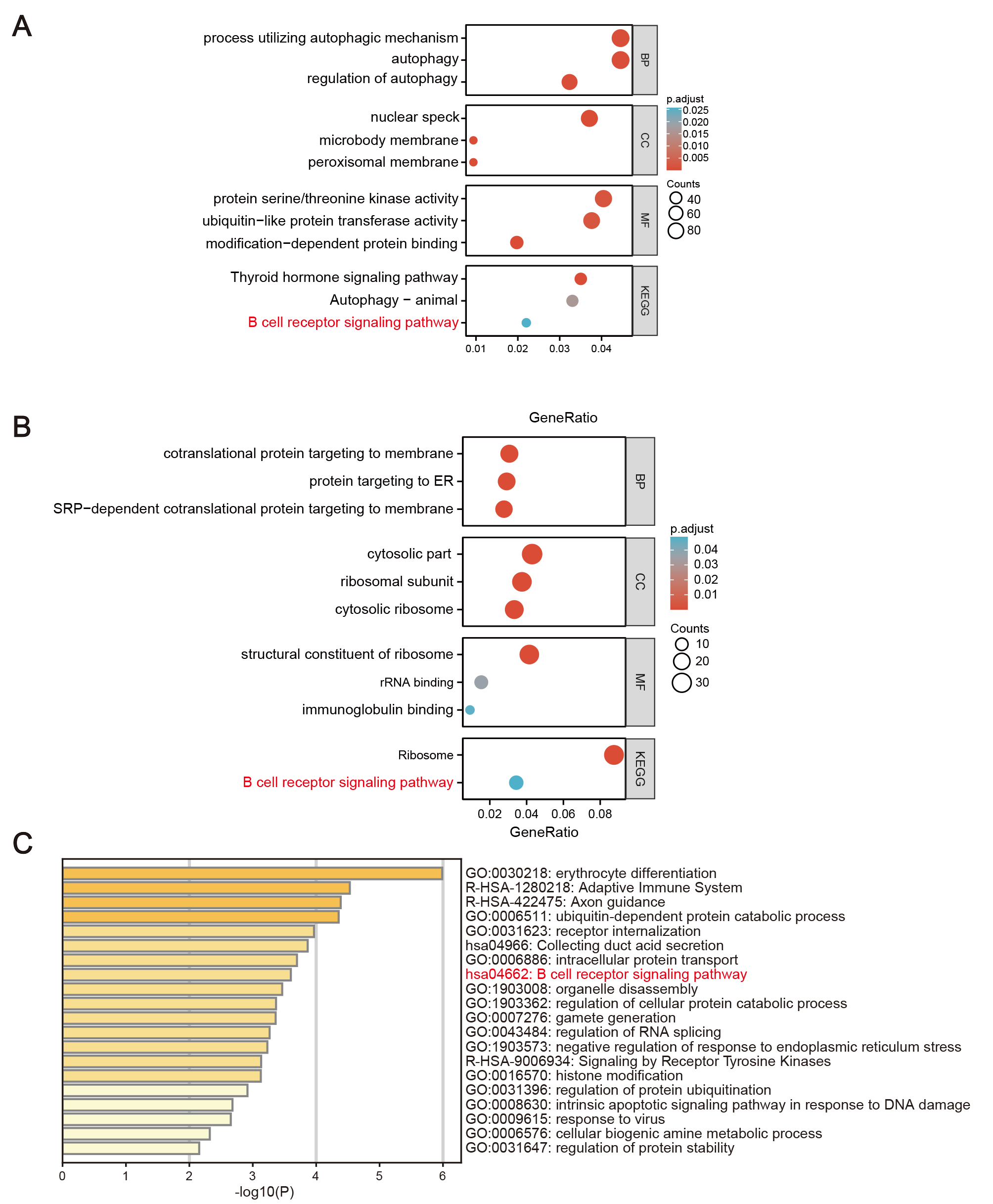


**Supplementary Figure 3.** The GO and KEGG analyses results of DEGs determined by different methods. (A) “Batch” method. (B) “RRA” method. (C) The intersection of “Batch” and “RRA” method.

**
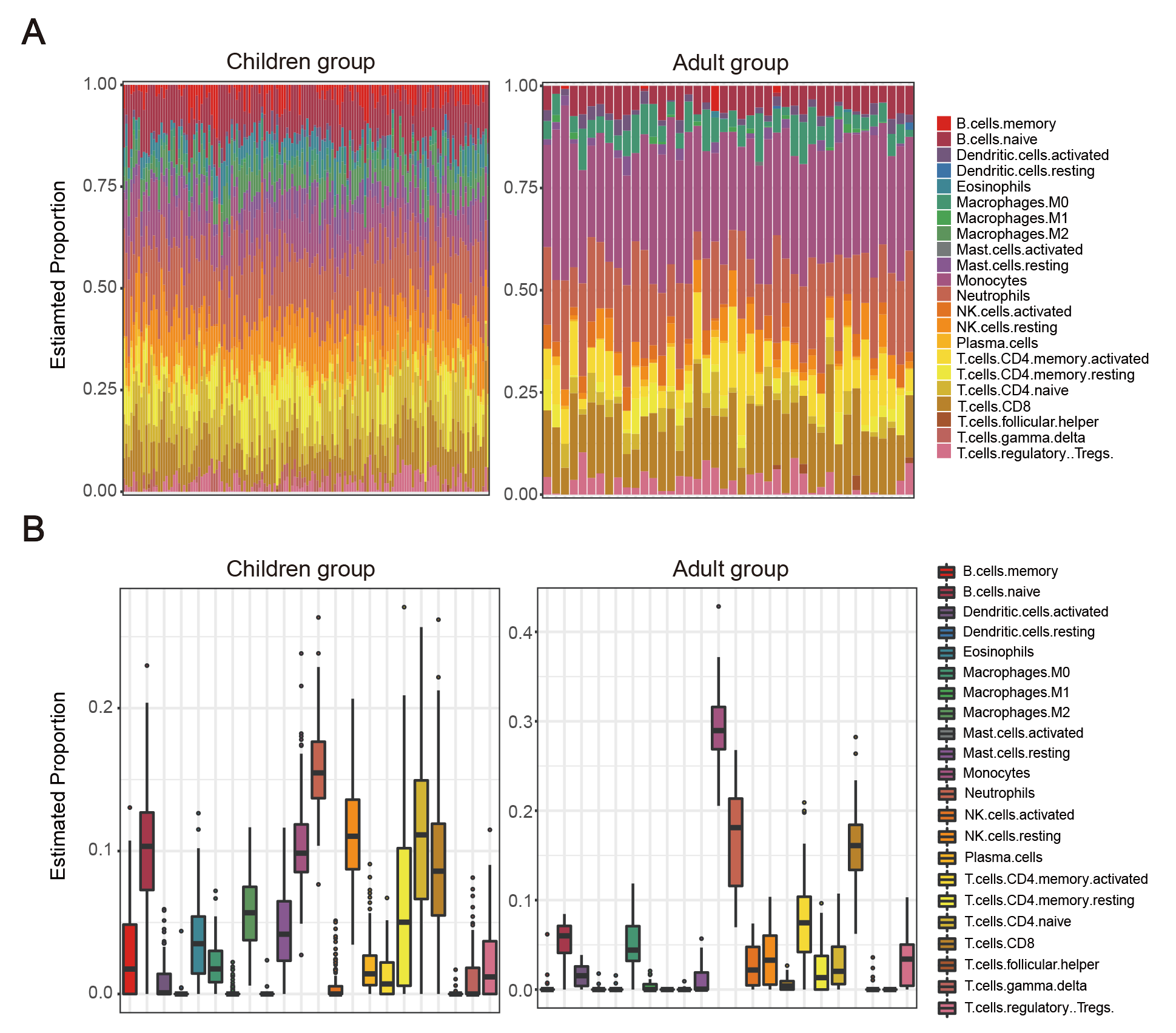
**

**Supplementary Figure 4.** The proportion of 22 types of immune cells in each sample from the child and adult groups. (A) The histogram of the composition of immune cells. (B) The box-plots of the composition of immune cells.


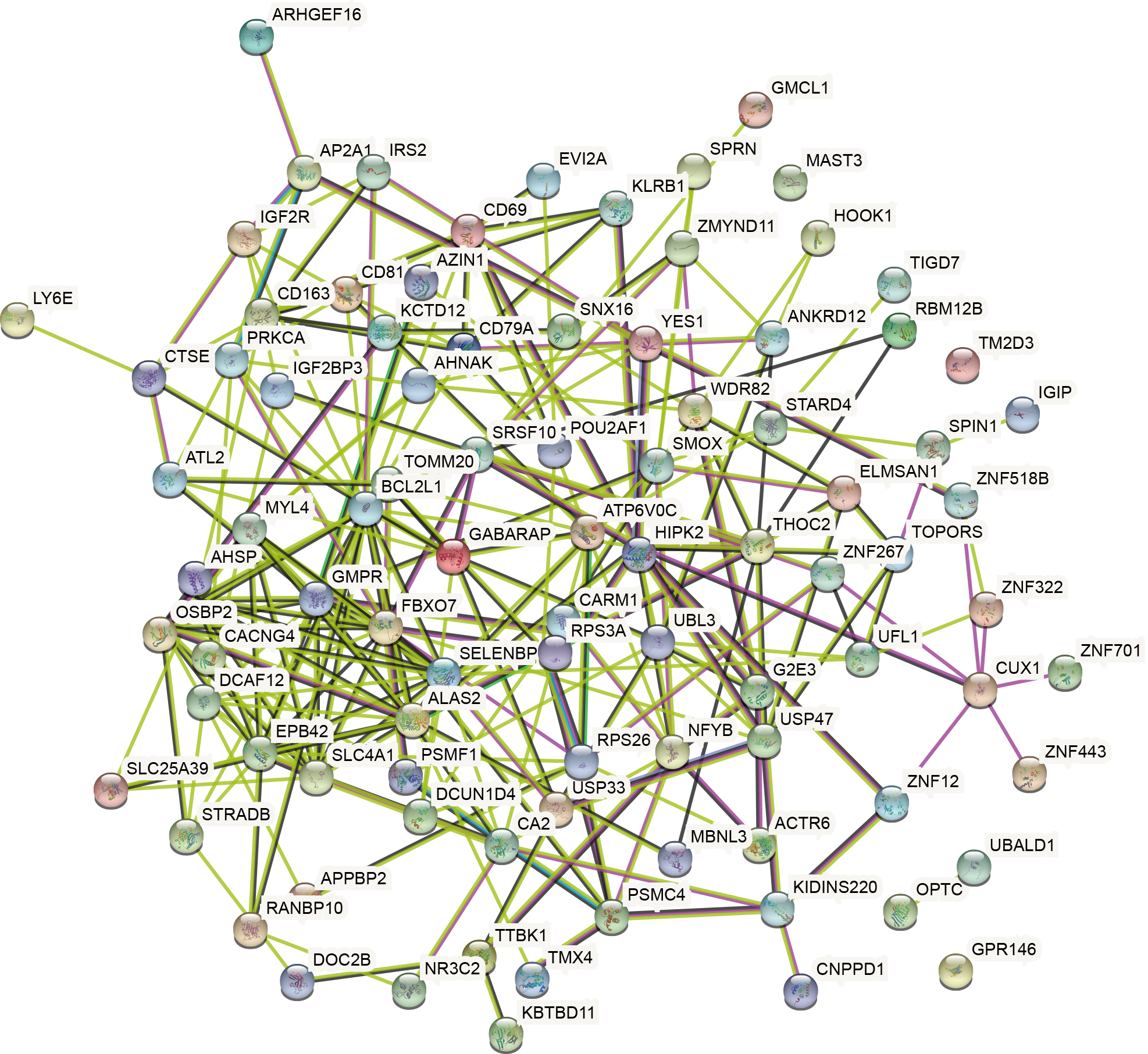


**Supplementary Figure 5.** PPI network analysis results of 95 consistently changing DEGs by STRING.

## Supplementary Tables

**Supplementary Table 1.** Basic information of selected ASD datasets.

| **GEO** | **Platform** | **Tissue** | **Samples (number)** | | | **Expression profiling** | **Attribute** | |
| --- | --- | --- | --- | --- | --- | --- | --- | --- |
|  |  | **Homo sapiens** | **ASD** | **TD** | **Total** | **Type** | **Group** | **Type** |
| GSE6575 | GPL570 | Whole blood | 35 | 12 | 47 | Array | Training | Child |
| GSE18123 | GPL570 | Whole blood | 66 | 33 | 99 | Array | Training | Child |
| GSE18123 | GPL6244 | Whole blood | 104 | 82 | 186 | Array | Training | Child |
| GSE42133 | GPL10558 | Whole blood | 91 | 56 | 147 | Array | Training | Child |
| GSE111175 | GPL10558 | Whole blood | 38 | 70 | 108 | Array | Validation | Child |
| GSE26415 | GPL6480 | Whole blood | 21 | 21 | 42 | Array | Adult group | |
| GSE89594 | GPL16699 | Whole blood | 26 | 30 | 56 | Array |  |  |

**Supplementary Table 2.** 95 consistently changing DEGs between RRA and Batch.

|  | **DEGs** |
| --- | --- |
| **Co-upregulated genes** | *RBM12B, KLRB1, HOOK1, USP9Y, IGIP, ZNF12, NR3C2, YES1, KBTBD11, KCTD12, MGC70870, IRS2, RPS3A, TIGD7, USP47, TOMM20, IGF2R, NFYB, TMX4, HIPK2, THOC2, PRKCA, ZNF518B, MAST3, MIDEAS, AHNAK, UBL3, SNHG5, KIDINS220, SPIN1, CD69, G2E3, ZMYND11, ZNF322, CA2, ZNF443, ZNF701, AZIN1, GVINP1, TOPORS, CUX1, SRSF10, ZNF267, STARD4, GMCL1, EVI2A, ACTR6, TM2D3, UFL1, DCUN1D4, WDR82, ANKRD12, CD163, APPBP2, USP33, SNX16, ATL2* |
| **Co-downregulated genes** | *GMPR, SELENBP1, DOC2B, CD79A, EPB42, ATP6V0C, PSMF1, LY6E, RPS26, IGF2BP3, ALAS2, SLC4A1, SLC25A39, PSMC4, DCAF12, MYL4, AHSP, RANBP10, OSBP2, GABARAP, CARM1, UBALD1, GPR146, AP2A1, STRADB, BCL2L1, FBXO7, OPTC, TTBK1, CACNG4, CNPPD1, CTSE, SMOX, CD81, POU2AF1, MBNL3, ARHGEF16, SPRN* |

**Supplementary Table 3.** 25 consistently changing DEGs between GSE26415 and GSE89594.

|  | **DEGs** |
| --- | --- |
| **Co-upregulated genes** | *CHRNB2, FAM47C, SOX21, TRRAP, CCDC169, NXPH4, TNFAIP3, LMBRD2, LRAT, H6PD, R3HDM2, SMAD3, FLJ11235, PATL1, MTCH2, TULP4, BCAT2* |
| **Co-downregulated genes** | *FAM165B, IDE, DNAAF2, PDZD7, GGT6, LOC729603, MOB1B, SMAD4* |

**Supplementary Table 4.** Summary of enrichment analysis in erythrocyte-related parameters and disorders in DisGeNET for the 95 consistently changing DEGs.

| **Description** | **Log*P*** | **Gene sets** |
| --- | --- | --- |
| Mean Corpuscular Hemoglobin | -5.2 | *AP2A1, GMPR, IGF2R, SLC4A1, IRS2, ANKRD12, FBXO7, DCAF12, CNPPD1, SMOX, MIDEAS* |
| Extramedullary Hematopoiesis Function | -4.8 | *CA2, EPB42, SLC4A1* |
| Red Blood Cell Distribution Width Determination | -4.8 | *GMPR, SLC4A1, IRS2, PSMF1, SRSF10, MAST3, OSBP2, SMOX, USP47, MIDEAS* |
| Anemia, Hemolytic | -2.9 | *ALAS2, CD81, EPB42, SLC4A1* |
| Mean Corpuscular Hemoglobin Concentration | -2.8 | *BCL2L1, GMPR, SLC4A1, FBXO7, RANBP10, ZNF322* |

**Supplementary Table 5.** Receiver operative characteristic curves of prob_min and prob_1se in the LASSO regression model.

|  | **Level** | | **Sensitivity** | **Specificity** | **95% CI** | **AUC** |
| --- | --- | --- | --- | --- | --- | --- |
|  | **TD** | **ASD** |  |  |  |  |
| prob_min | 0.22 ± 2.42 | -1.53 ± 1.17 | 0.58 | 0.92 | 0.739-0.888 | 0.814 |
| prob_1se | -0.52 ± 0.48 | -1.01 ± 0.50 | 0.84 | 0.68 | 0.692-0.861 | 0.776 |

**Abbreviations**

| *RBM12B* | RNA binding motif protein 12B |
| --- | --- |
| *KLRB1* | killer cell lectin like receptor B1 |
| *HOOK1* | hook microtubule tethering protein 1 |
| *USP9Y* | ubiquitin specific peptidase 9 Y-linked |
| *IGIP* | IgA inducing protein |
| *ZNF12* | zinc finger protein 12 |
| *NR3C2* | nuclear receptor subfamily 3 group C member 2 |
| *YES1* | YES proto-oncogene 1, Src family tyrosine kinase |
| *KBTBD11* | kelch repeat and BTB domain containing 11 |
| *KCTD12* | potassium channel tetramerization domain containing 12 |
| *MGC70870* | C-terminal binding protein 2 pseudogene |
| *IRS2* | insulin receptor substrate 2 |
| *RPS3A* | ribosomal protein S3A |
| *TIGD7* | tigger transposable element derived 7 |
| *USP47* | ubiquitin specific peptidase 47 |
| *TOMM20* | translocase of outer mitochondrial membrane 20 |
| *IGF2R* | insulin like growth factor 2 receptor |
| *NFYB* | nuclear transcription factor Y subunit beta |
| *TMX4* | thioredoxin related transmembrane protein 4 |
| *HIPK2* | homeodomain interacting protein kinase 2 |
| *THOC2* | THO complex subunit 2 |
| *PRKCA* | protein kinase C alpha |
| *ZNF518B* | zinc finger protein 518B |
| *MAST3* | microtubule associated serine/threonine kinase 3 |
| *MIDEAS* | mitotic deacetylase associated SANT domain protein |
| *AHNAK* | AHNAK nucleoprotein |
| *UBL3* | ubiquitin like 3 |
| *SNHG5* | small nucleolar RNA host gene 5 |
| *KIDINS220* | kinase D interacting substrate 220 |
| *SPIN1* | spindlin 1 |
| *CD69* | CD69 molecule |
| *G2E3* | G2/M-phase specific E3 ubiquitin protein ligase |
| *ZMYND11* | zinc finger MYND-type containing 11 |
| *ZNF322* | zinc finger protein 322 |
| *CA2* | carbonic anhydrase 2 |
| *ZNF443* | zinc finger protein 443 |
| *ZNF701* | zinc finger protein 701 |
| *AZIN1* | antizyme inhibitor 1 |
| *GVINP1* | GTPase, very large interferon inducible pseudogene 1 |
| *TOPORS* | TOP1 binding arginine/serine rich protein, E3 ubiquitin ligase |
| *CUX1* | cut like homeobox 1 |
| *SRSF10* | serine and arginine rich splicing factor 10 |
| *ZNF267* | zinc finger protein 267 |
| *STARD4* | StAR related lipid transfer domain containing 4 |
| *GMCL1* | germ cell-less 1, spermatogenesis associated |
| *EVI2A* | ecotropic viral integration site 2a |
| *ACTR6* | actin related protein 6 |
| *TM2D3* | TM2 domain containing 3 |
| *UFL1* | UFM1 specific ligase 1 |
| *DCUN1D4* | defective in cullin neddylation 1 domain containing 4 |
| *WDR82* | WD repeat domain 82 |
| *ANKRD12* | ankyrin repeat domain 12 |
| *CD163* | CD163 molecule |
| *APPBP2* | amyloid beta precursor protein binding protein 2 |
| *USP33* | ubiquitin specific peptidase 33 |
| *SNX16* | sorting nexin 16 |
| *ATL2* | atlastin GTPase 2 |
| *GMPR* | guanosine monophosphate reductase |
| *SELENBP1* | selenium binding protein 1 |
| *DOC2B* | double C2 domain beta |
| *CD79A* | CD79a molecule |
| *EPB42* | erythrocyte membrane protein band 4.2 |
| *ATP6V0C* | ATPase H+ transporting V0 subunit c |
| *PSMF1* | proteasome inhibitor subunit 1 |
| *LY6E* | lymphocyte antigen 6 family member E |
| *RPS26* | ribosomal protein S26 |
| *IGF2BP3* | insulin like growth factor 2 mRNA binding protein 3 |
| *ALAS2* | 5'-aminolevulinate synthase 2 |
| *SLC4A1* | solute carrier family 4 member 1 |
| *SLC25A39* | solute carrier family 25 member 39 |
| *PSMC4* | proteasome 26S subunit, ATPase 4 |
| *DCAF12* | DDB1 and CUL4 associated factor 12 |
| *MYL4* | myosin light chain 4 |
| *AHSP* | alpha hemoglobin stabilizing protein |
| *RANBP10* | RAN binding protein 10 |
| *OSBP2* | oxysterol binding protein 2 |
| *GABARAP* | GABA type A receptor-associated protein |
| *CARM1* | coactivator associated arginine methyltransferase 1 |
| *UBALD1* | UBA-like domain containing 1 |
| *GPR146* | G protein-coupled receptor 146 |
| *AP2A1* | adaptor related protein complex 2 subunit alpha 1 |
| *STRADB* | STE20 related adaptor beta |
| *BCL2L1* | BCL2 like 1 |
| *FBXO7* | F-box protein 7 |
| *OPTC* | opticin |
| *TTBK1* | tau tubulin kinase 1 |
| *CACNG4* | calcium voltage-gated channel auxiliary subunit gamma 4 |
| *CNPPD1* | cyclin Pas1/PHO80 domain containing 1 |
| *CTSE* | cathepsin E |
| *SMOX* | spermine oxidase |
| *CD81* | CD81 molecule |
| *POU2AF1* | POU class 2 homeobox associating factor 1 |
| *MBNL3* | muscleblind like splicing factor 3 |
| *ARHGEF16* | Rho guanine nucleotide exchange factor 16 |
| *SPRN* | shadow of prion protein |
| *CHRNB2* | cholinergic receptor nicotinic beta 2 subunit |
| *FAM47C* | family with sequence similarity 47 member C |
| *SOX21* | SRY-box transcription factor 21 |
| *TRRAP* | transformation/transcription domain associated protein |
| *CCDC169* | coiled-coil domain containing 169 |
| *NXPH4* | neurexophilin 4 |
| *TNFAIP3* | TNF alpha induced protein 3 |
| *LMBRD2* | LMBR1 domain containing 2 |
| *LRAT* | lecithin retinol acyltransferase |
| *H6PD* | hexose-6-phosphate dehydrogenase/glucose 1-dehydrogenase |
| *R3HDM2* | R3H domain containing 2 |
| *SMAD3* | SMAD family member 3 |
| *FLJ11235* | hypothetical protein FLJ11235 |
| *PATL1* | PAT1 homolog 1, processing body mRNA decay factor |
| *MTCH2* | mitochondrial carrier 2 |
| *TULP4* | TUB like protein 4 |
| *BCAT2* | branched chain amino acid transaminase 2 |
| *FAM165B* | small integral membrane protein 11 |
| *IDE* | insulin degrading enzyme |
| *DNAAF2* | dynein axonemal assembly factor 2 |
| *PDZD7* | PDZ domain containing 7 |
| *GGT6* | gamma-glutamyltransferase 6 |
| *LOC729603* | Calcineurin-like EF-hand protein 1 pseudogene |
| *MOB1B* | MOB kinase activator 1B |
| *SMAD4* | SMAD family member 4 |
